# The Effect of Cognitive Training After Heart Valve Surgery: A Systematic Review

**DOI:** 10.3390/jcm15010370

**Published:** 2026-01-04

**Authors:** You Gyoung Yi, Younji Kim, Daegil Kwon, Seoyon Yang, Min Cheol Chang

**Affiliations:** 1 Department of Rehabilitation Medicine, College of Medicine, Ewha Womans University, Seoul 03760, Republic of Korea; lyk861124@gmail.com (Y.G.Y.); yunji0114@naver.com (Y.K.); 2 Department of Physical Medicine and Rehabilitation, Daegu Myungsung Medical Center, Daegu 42668, Republic of Korea; cateyesn@naver.com; 3 Department of Physical Medicine and Rehabilitation, College of Medicine, Yeungnam University, Daegu 42415, Republic of Korea

**Keywords:** cognitive therapy, cognition disorders, heart valve surgical procedures, cognition, review

## Abstract

**Background**: Neurocognitive complications are common after cardiac surgery, and postoperative cognitive decline remains a clinically relevant concern in patients undergoing heart valve surgery. This impairment may persist over time and negatively affect quality of life and increase mortality risk. This review aimed to explore the potential benefits of cognitive training in patients after heart valve surgery. **Methods:** We systematically searched PubMed, Embase, Cochrane Library, and Scopus to identify articles published from database inception to 19 June 2025. Studies that investigated the effects of cognitive training in patients who underwent heart valve surgery were included. **Results:** A total of 1506 articles were identified. After title and abstract screening, 1476 articles not meeting the inclusion criteria were excluded. Thirty full-text articles were assessed for eligibility, of which four studies were ultimately included in this review. Cognitive training was found to significantly improve cognitive outcomes and health-related quality of life, with benefits sustained for up to 12 months postoperatively. **Conclusions:** This review highlights cognitive training as a promising, feasible, and effective intervention for preserving cognitive function in patients following heart valve surgery. By enhancing neuroplasticity, cognitive training may prevent or mitigate cognitive decline across multiple domains. Further large-scale studies are warranted to confirm the efficacy of this treatment in this patient population.

## 1. Introduction

Neurocognitive complications occur in approximately 33–80% of patients after cardiac surgery and are associated with increased postoperative morbidity and mortality, greater economic burden, and prolonged hospital stay [1]. Neurocognitive complications include delirium, dementia, and postoperative cognitive decline (POCD). POCD is defined as a decline in postoperative cognitive performance in at least 20% of the objectively assessed cognitive domains, such as selective attention, verbal memory, and word fluency [2]. POCD can lead to persistent long-term cognitive impairment and reduced quality of life; therefore, early detection and ongoing monitoring are essential.

Patients undergoing heart valve procedures—including aortic valve replacement (AVR), transcatheter AVR (TAVR), mitral valve replacement (MVR), and mitral valve repair—are particularly susceptible to cognitive decline, especially during the early postoperative period [3,4]. The etiology of cognitive decline after heart valve surgery is multifactorial, involving both perioperative and patient-related factors [5]. These factors may contribute to cerebral vulnerability and subsequent cognitive impairment after surgery [6].

Cognitive training refers to structured repetitive exercises designed to target specific cognitive functions and enhance performance in one or more cognitive domains [7]. Cognitive training can improve both cognitive ability and everyday functioning, potentially contributing to a better quality of life in older adults [8]. Furthermore, recent meta-analyses have reported that cognitive training after cardiac surgery significantly reduces POCD incidence and improves postoperative cognitive function [9,10]. These findings support the growing evidence that targeted cognitive interventions may help mitigate surgery-related cognitive decline. Consequently, the role of cognitive training is gaining increasing attention in the context of recovery following heart valve surgery.

In this review, we aimed to examine the effects of cognitive training in patients who underwent heart valve surgery.

## 2. Materials and Methods

A systematic review was conducted to synthesize the available evidence regarding the effects of cognitive training in patients who underwent heart valve surgery.

### 2.1. Search Strategy

This review was performed in accordance with the Preferred Reporting Items for Systematic Reviews and Meta-Analyses (PRISMA) guidelines [11]. We comprehensively searched the PubMed, Embase, Cochrane Library, and Scopus databases for relevant articles published from database inception to 19 June 2025. The search strategy combined controlled vocabulary terms (MeSH and Emtree) and free-text keywords related to heart valve surgery (e.g., aortic valve replacement, TAVR, surgical AVR, mitral valve repair, and mitral valve replacement) and cognitive interventions (e.g., cognitive training, cognitive rehabilitation, neuropsychological rehabilitation, and memory training). Detailed search strategies for each database are provided in the Supplementary Materials (Supplementary File S1).

#### Review Registration

This systematic review has been registered in the Open Science Framework (OSF). The registration DOI is 10.17605/OSF.IO/5GSH. Post-registration was completed to ensure methodological transparency and prevent unintended duplication of similar ongoing reviews.

### 2.2. Study Selection

Studies investigating the effects of cognitive training in patients who underwent heart valve surgery were included. The inclusion criteria were as follows: (1) studies involving patients who underwent heart valve surgery; (2) studies in which these patients received cognitive training postoperatively; and (3) studies using validated assessment tools to measure cognitive changes following training. The exclusion criteria were as follows: (1) studies unrelated to heart valve surgery and cognitive training; (2) animal studies, case reports, reviews, commentaries, and letters; and (3) studies with insufficient or unreported outcomes. Two independent reviewers (S.Y.Y. and Y.K.Y.) screened titles and abstracts to exclude irrelevant articles. Full-text articles were subsequently reviewed for final inclusion. Any disagreements were resolved through discussion, with the involvement of a third reviewer (M.C.C.) when necessary.

### 2.3. Data Extraction

Two reviewers (S.Y.Y. and Y.G.Y.) independently extracted data using a standardized data collection form. For each eligible study, the following information was recorded: (1) first author’s name, (2) year of publication, (3) study design, (4) number of participants, (5) characteristics of the intervention group, (6) characteristics of the control group(s), (7) duration of the intervention, (8) follow-up period, (9) outcome assessment methods, and (10) main findings.

### 2.4. Quality Assessment

For randomized controlled trials (RCTs), the methodological quality was evaluated using the Cochrane Collaboration’s Risk of Bias tool version 1. This tool assesses several predefined domains, including random sequence generation, allocation concealment, blinding of participants and personnel, blinding of outcome assessment, incomplete outcome data, selective reporting, and other potential sources of bias. Risk of bias judgments were assigned as low, high, or unclear in accordance with the criteria outlined in the Cochrane Handbook. Random sequence generation and allocation concealment were rated as low risk when explicitly described. Blinding of participants and personnel was rated as high risk in all studies owing to the nature of cognitive training interventions. Blinding of outcome assessment was rated as low risk when objective neuropsychological assessments were performed by blinded assessors and rated as unclear risk when outcomes relied primarily on self-reported measures. Incomplete outcome data were rated as unclear risk when attrition rates were higher at long-term follow-up without sufficient justification.

## 3. Results

**Figure 1:**
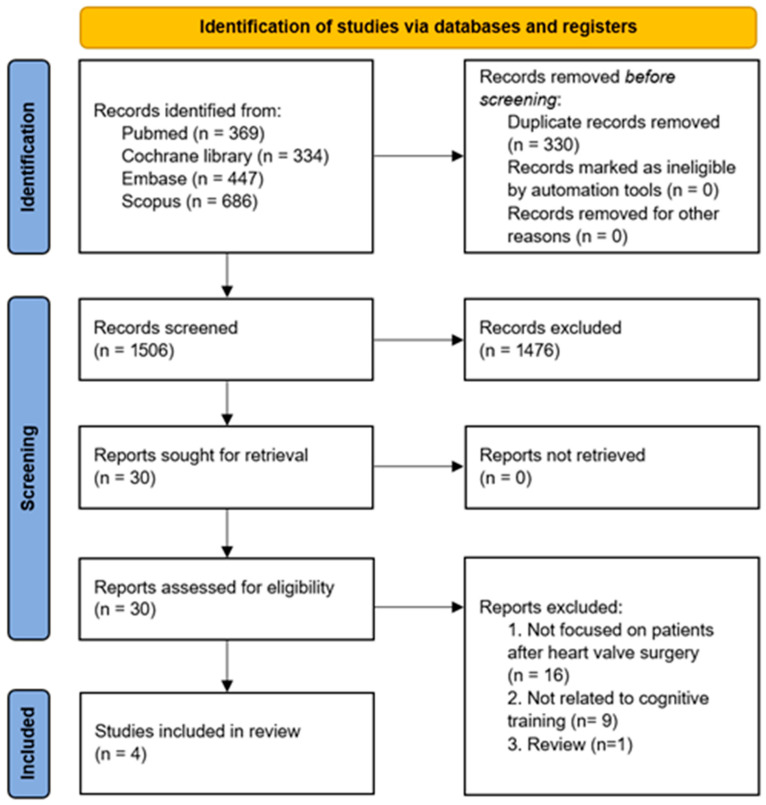
Flow diagram of the study selection process.

A total of 1506 articles were identified through the initial search. After title and abstract screening, 1476 articles not meeting the inclusion criteria were excluded. Upon assessing 30 full-text articles for eligibility, 26 were excluded for the following reasons: 16 did not involve patients who underwent heart valve surgery, 9 were not related to cognitive training, and 1 was a review article. Ultimately, four articles investigating the effects of cognitive training after heart valve surgery were included in this review (Figure 1). The characteristics of the included studies are summarized in Table 1.

**Figure 2:**
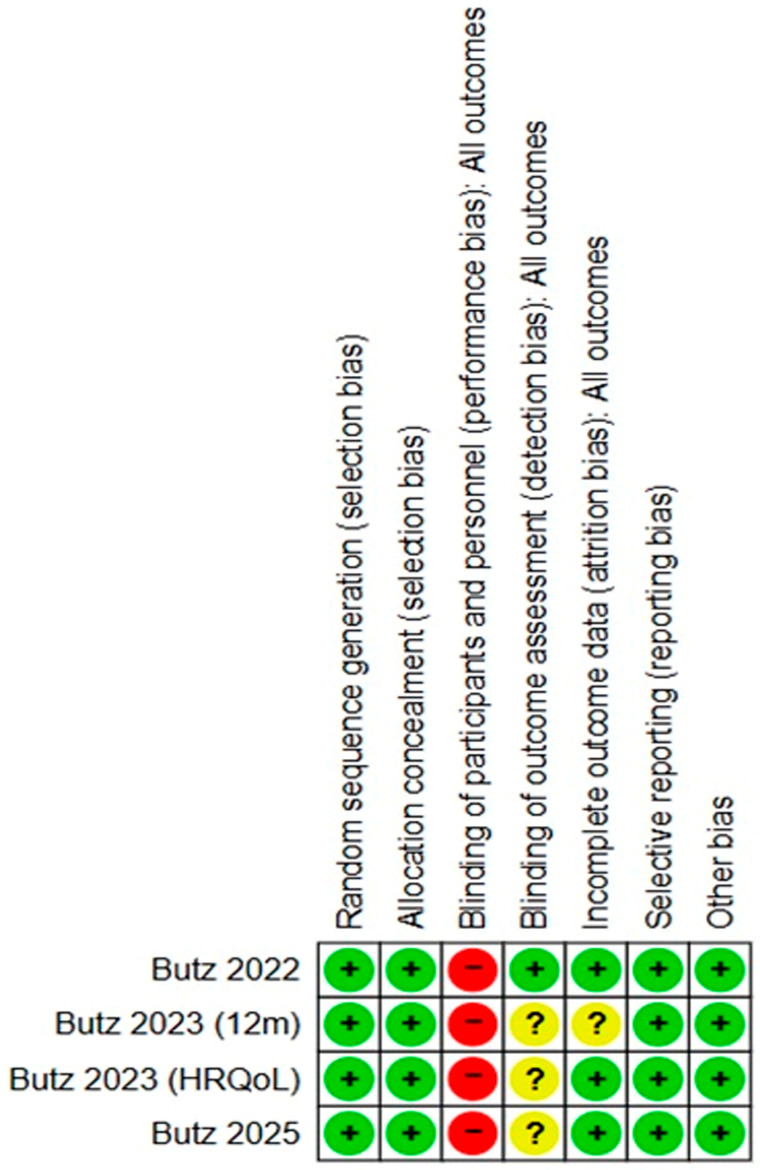
Results of quality assessment of the included studies. +, low risk of bias; −, high risk of bias; ?, unclear risk of bias [12,13,14,15].

Of the four RCTs included in this review, the overall risk of bias was low, with all studies providing adequate descriptions of random sequence generation and allocation concealment. All four trials were judged as having a high risk of performance bias, because the cognitive-training intervention precluded blinding of participants and personnel. For blinding of outcome assessment, one study [12] demonstrated a low risk of bias through the use of blinded neuropsychological assessment, whereas the remaining three studies [13,14,15] were rated as unclear because outcome evaluation relied primarily on self-reported outcomes (health-related quality of life [HRQoL] questionnaires, Cognitive Failure Questionnaire [CFQ]). Regarding incomplete outcome data, three trials [12,13,15] had a low risk of bias, whereas one study [14] was rated as having an unclear risk of bias owing to higher dropout rates at the 12-month follow-up and limited justification. All studies were judged as having a low risk of selective reporting and other potential sources of bias (Figure 2). Across the included RCTs, POCD was defined using a consistent operational criterion (i.e., a decline of ≥1 SD in ≥20% of objectively assessed cognitive domains) based on a common multidomain neuropsychological test battery.

The first study evaluating the effects of cognitive training after heart valve surgery was published in 2022 by Butz et al. [12]. This RCT assessed the impact of early postoperative cognitive training in older patients undergoing elective AVR or MVR. Participants in the control group (*n* = 44) received standard inpatient cardiac rehabilitation including endurance training, strength training, and respiratory exercises. The cognitive training group (*n* = 37) received the same rehabilitation program in addition to a structured paper-and-pencil-based cognitive training program targeting multiple domains, including attention, working memory, and verbal fluency. Training sessions were administered 6 days per week for 3 weeks. Neuropsychological assessments were performed before surgery, at discharge, and 3 months after discharge. The cognitive training group demonstrated significantly lower rates of POCD than the control group both at discharge (19% vs. 50%) and the 3-month follow-up (6% vs. 29%). Additionally, the rate of postoperative cognitive improvement (POCI) was significantly higher in the cognitive training group at 3 months (36% vs. 15%). These findings indicate that early cognitive training is effective in preventing and reducing POCD occurrence.

Butz et al. also investigated the impact of postoperative cognitive training on HRQoL in patients after heart valve surgery [13]. A total of 60 older patients who underwent aortic or mitral valve replacement or reconstruction completed the trial (cognitive training group, *n* = 31; control group, *n* = 29). HRQoL outcomes were assessed using the 36-Item Short Form Health Survey (SF-36) and the CFQ administered preoperatively and 3 months postoperatively. The SF-36 evaluates eight domains of health-related functioning, including physical functioning, emotional well-being, and general health. The CFQ measures self-reported cognitive failures in everyday life, such as difficulties related to memory, attention, action, and perception [13]. The cognitive training group received approximately 15 days of paper-and-pencil-based cognitive training, in addition to standard inpatient cardiac rehabilitation, which was also provided to the control group. The results demonstrated that the cognitive training group experienced significantly greater improvements in global HRQoL, particularly in domains related to emotional problems, energy and fatigue, social functioning; overall SF-36 scores; and perceived health change than to the control group. These findings suggest that cognitive training may enhance postoperative quality of life, especially in emotional and social domains, in patients after heart valve surgery.

In a 2023 follow-up RCT, Butz et al. reported the long-term effects of postoperative cognitive training on cognitive function and HRQoL in patients who underwent heart valve surgery [14]. The control group (*n* = 28) received standard inpatient cardiac rehabilitation, whereas the cognitive training group (*n* = 30) received the same rehabilitation program with the addition of paper-based cognitive training targeting multiple cognitive domains, including memory, attention, and executive function. Both groups underwent treatment for 3 weeks. The SF-36 and CFQ were used to assess HRQoL and cognitive failure in daily life before surgery and at the 12-month follow-up. At 12 months, the cognitive training group demonstrated significantly greater improvements in HRQoL, particularly in domains related to role limitations due to physical and emotional health, pain, and overall health perception. Improvements in visual recognition memory were also observed, and the incidence of POCD was lower compared with that in the control group (11% vs. 22%). These findings suggest that early postoperative cognitive training provides both short-term and sustained benefits for HRQoL and cognitive function for up to 1 year after heart valve surgery.

The most recent RCT published in 2025 by Butz et al. investigated whether brain lesions—specifically white matter lesions (WML) and postoperative subclinical cerebral ischemia (SCI), both recognized risk factors for POCD—influence the effectiveness of cognitive training in older patients undergoing heart valve surgery [15]. All participants received standard cardiac rehabilitation beginning 1 week postoperatively and were randomized into a cognitive training group (*n* = 18) and control group (*n* = 21). The cognitive training group received an additional structured paper-and-pencil cognitive training program administered 6 days per week for 3 weeks. Cranial magnetic resonance imaging (MRI) was performed 6–10 days postoperatively to identify preexisting WML and SCI. Cognitive function, depressive symptoms, and HRQoL were assessed using neuropsychological tests, the Hospital Anxiety and Depression Scale, and the SF-36 questionnaire. At the 3-month follow-up, the cognitive training group showed significant improvements in global cognition, depressive symptoms, and several SF-36 domains (including the mental component summary), even after adjustment for the presence and severity of WML and SCI. Subgroup analyses revealed that patients with moderate to severe WML or SCI benefited from cognitive training, particularly in global cognition, emotional well-being, and general health. These findings indicate that cognitive training may be an effective intervention for preserving cognitive function and improving HRQoL, even in high-risk patients with cerebral lesions after heart valve surgery.

## 4. Discussion

The findings of the included studies suggest that early cognitive training after heart valve surgery is a feasible and effective strategy for preventing cognitive decline and enhancing cognitive function. These benefits were observed in the short term (3 months after surgery) and appeared to be sustained over longer follow-up periods of up to 1 year postoperatively.

Cognitive decline observed in the early postoperative period after heart valve surgery may be explained by several pathophysiological mechanisms proposed in previous studies [13]. Perioperative risk factors, including preexisting mild cognitive impairment, anxiety, and depression may increase vulnerability to POCD after heart valve surgery [16,17]. In addition, perioperative risk factors related to anesthesia and surgery, such as cerebral microembolization and neuroinflammatory responses, are thought to play a central role in POCD development [18]. Furthermore, impaired cerebral autoregulation, intraoperative cerebral hypoperfusion, and surgery-induced systemic and neuroinflammation may exacerbate neuronal injury and synaptic dysfunction, thereby contributing to postoperative cognitive impairment [6]. From a broader brain–heart axis perspective, systemic inflammatory responses triggered by cardiac surgery may interact bidirectionally with central nervous system function. In this context, cognitive training may exert beneficial effects not only through task-specific cognitive engagement but also through top-down neural activation, potentially modulating neuroinflammatory pathways and autonomic regulation. Given these mechanisms, early postoperative cognitive training has been suggested as a potential strategy to mitigate POCD and facilitate cognitive recovery in this patient population.

The present findings are consistent with emerging clinical evidence indicating that postoperative cognitive training attenuates cognitive decline in patients after cardiac surgery. Zhang et al. [9] reported a markedly lower POCD rate in patients receiving cognitive training, corroborating the results of Zhao et al. [10]. Likewise, Butz et al. demonstrated in an RCT that cognitive training significantly reduced early postoperative cognitive dysfunction after heart valve surgery [12], with sustained improvements in patient-reported outcomes at 12 months postoperatively [14]. Collectively, these studies reinforce the conclusion that postoperative cognitive training can effectively reduce POCD and improve recovery in patients after heart valve surgery.

Heart valve surgery prevents further cardiac deterioration and may lead to acute improvements in cerebral perfusion. Preoperatively, patients with severe valvular disease (e.g., aortic stenosis) often experience chronic cerebral hypoperfusion, which contributes to cognitive impairment [19]. Surgical correction restores cardiac output and may lead to cognitive improvement by increasing cerebral blood flow [20]. We propose that postoperative cognitive training can capitalize on this hemodynamic recovery. Increased cerebral perfusion after surgery may enhance the effectiveness of cognitive training by improving the delivery of nutrients and oxygen required for neuroplastic processes, such as synaptic strengthening and dendritic growth. This idea is supported by evidence indicating that cognitive training interventions can augment cerebral blood flow in older adults [20], suggesting a reciprocal reinforcement in which improved circulation facilitates training-induced brain plasticity, while engaging in mental training can further support cerebral perfusion.

One possible mechanism underlying the effects of cognitive training is the enhancement of neuroplasticity. Neuroplasticity refers to the ability of the brain to modify its structure and function through formation, strengthening, weakening, or elimination of neural connections [21]. This process can be influenced by environmental stimuli, experiences, or learning, and may occur in response to both adaptive and maladaptive changes [22]. Cognitive resilience can be promoted through neuroplastic adaptation via multiple mechanisms including synaptic strengthening, compensatory processes, and network reorganization [23]. The timing and intensity of an intervention are critical factors in driving neuroplastic changes related to brain repair and functional recovery [24]. In this context, both the timing of delivery and nature of the intervention are key determinants of how effectively neuroplasticity is induced.

Targeted interventions such as cognitive training may help prevent or slow cognitive decline by enhancing neuroplasticity and supporting the brain’s capacity to reorganize and form new neural connections after heart valve surgery [25]. Importantly, the early postoperative period may represent a window of heightened cerebral vulnerability but also offers the potential for increased neuroplasticity during which structured cognitive engagement can promote top-down reactivation and functional reintegration of disrupted cognitive networks following surgery-related cerebral stress [26]. Evidence indicates that cognitive training significantly improves recognition, executive function (including working memory and processing speed), and overall cognitive performance compared with control treatment [8]. Additionally, it has been associated with improvements in memory tasks and self-reported cognitive function. Taken together, these findings support a conceptual framework in which cardiac surgery-related cerebral stress transiently disrupts cognitive networks, whereas timely and targeted cognitive training facilitates adaptive reorganization, thereby contributing to cardiac–cognitive recovery in patients undergoing heart valve surgery.

Cognitive training also offers several practical advantages. Once patients are appropriately instructed, training can be performed independently without the need for intensive supervision [12]. Moreover, paper-and-pencil-based cognitive training is inexpensive and may reduce the economic burden associated with postoperative care [12]. Therefore, early cognitive training is feasible and easily applicable for patients at risk of postoperative cognitive dysfunction and may represent a promising adjunctive intervention following heart valve surgery, although further confirmation is required.

We acknowledge the growing interest in preoperative cognitive “prehabilitation” as a strategy to mitigate postoperative cognitive decline. Recent evidence suggests that this approach has merit; a 2024 meta-analysis of RCTs reported that cognitive training administered before surgery was associated with a significantly lower incidence of POCD than that observed in control groups [10]. Similarly, an RCT by Jiang et al. observed that 10 days of preoperative cognitive exercise reduced delirium occurrence by approximately 57% in older adults undergoing coronary artery bypass grafting [27]. These findings indicate that enhancing patients’ cognitive reserve before surgery may improve postoperative neurocognitive outcomes. While prehabilitation may help prevent cognitive decline, postoperative training aims to rehabilitate and enhance recovery of cognitive function after surgery. Both strategies are complementary, and future studies should clarify the optimal timing and integration of cognitive training across the perioperative period.

In patients undergoing heart valve surgery, cognitive training plays a critical role in preserving cognitive function and maintaining quality of life. Butz et al. reported parallel improvements in cognitive performance and HRQoL following cognitive training, with effects persisting for up to 12 months postoperatively [14]. Improvements in working memory were associated with improved emotion regulation, contributing to fewer role limitations related to emotional problems. Furthermore, improvements in executive functions, including planning, volition, goal-directed behavior, and inhibitory control, led to increased physical activity. These findings suggest that cognitive training can improve both cognitive outcomes and overall quality of life, emphasizing the importance of addressing cognitive health in patients after heart valve surgery.

Importantly, although cognitive training supports short- and mid-term recovery, its long-term effects on cognitive trajectories and dementia risk remain uncertain. The longest available follow-up period was 12 months postoperatively [14], during which participants receiving cognitive training demonstrated superior cognitive function and quality of life compared with control participants. However, to date, no study has evaluated whether such interventions influence the incidence of persistent neurocognitive disorders, such as mild cognitive impairment or dementia, over extended periods. It is therefore plausible that cognitive training facilitates recovery toward preoperative baseline without modifying the underlying trajectory of age-related cognitive decline.

The mode of delivery may also influence patient engagement, particularly in older adults. All the reviewed studies employed paper-based training methods, which are generally easier for older patients to adopt. However, digital platforms, such as app-based or virtual reality interventions, may offer advantages such as adaptive difficulty, performance feedback, and gamification, provided that interface usability is optimized for aging users. Future research should directly compare analog and digital formats in this population to assess feasibility, adherence, and cognitive outcomes.

Individual differences such as cognitive reserve may also influence how patients respond to cognitive training. Cognitive reserve, which is often indexed by factors such as educational attainment, occupational complexity, and cognitively enriching activities, has been shown to moderate susceptibility to cognitive decline. Although the studies in this review did not stratify outcomes based on cognitive reserve, previous work supports its relevance. Kainz et al. [28] found that patients with higher cognitive reserves had a significantly lower risk of early POCD after surgery. This suggests that those with greater reserves may experience greater resilience and faster recovery. Conversely, individuals with lower reserves might derive the greatest benefit from cognitive training, as it could help compensate for reduced baseline adaptability. Future studies should assess cognitive reserve and examine its interaction with training outcomes.

This study has several limitations. First, the number of included studies was small, and the studies were primarily conducted by the same research group, which might introduce bias and limit the generalizability of the findings to a broader population. Additionally, some of the included publications might have presented different analyses or follow-up reports derived from the same or partially overlapping trial populations, which could potentially overestimate the observed effects. However, the definition of POCD and neuropsychological assessment framework were consistent across the included trials, thereby minimizing heterogeneity related to the outcome definition. Second, individual studies had relatively small sample sizes. Third, the assessments of cognitive function and HRQoL were limited to a narrow range of measurement tools. Given these methodological considerations, including potential population overlap and heterogeneity in outcomes and follow-up durations, a quantitative meta-analysis was not feasible, and the results should be interpreted as a qualitative synthesis. Despite these limitations, this review provides valuable insight into the potential effects of cognitive training before and after heart valve surgery. Future multicenter studies with larger sample sizes, more diverse patient populations, and more comprehensive assessment strategies are warranted.

## 5. Conclusions

This review highlights the potential benefits of cognitive training as a feasible and effective preventive intervention to preserve cognitive function in patients undergoing heart valve surgery. Cognitive training may prevent or mitigate POCD by enhancing neuroplasticity and improving cognitive function across multiple domains. Additionally, improvements in health-related quality of life have been observed, with sustained positive effects lasting up to 12 months after surgery. Considering the high prevalence and clinical impact of cognitive dysfunction, further large-scale studies are warranted to evaluate the efficacy of cognitive training in this patient population.

## Figures and Tables

**Table 1:** Characteristics of included studies.

No.	Author	Year	Study Design	Number of Participants (Treatment vs. Control)	Type of Intervention	Control Group	Treatment Duration	Follow-Up Period	Evaluation Tool	**Effect Size**	**Results**
1	Butz et al. [12]	2022	RCT	60 (31 vs. 29)	Paper-and-pencil cognitive training	No cognitive training (standard cardiac rehabilitation)	36 min/day, 6 days/week for 3 weeks	3 months	Neuropsychological test battery (visual/verbal memory, executive function, attention)	POCD incidence: OR = 4.29 (discharge); OR = 6.46 (3 months)	The cognitive training group showed significantly lower rates of POCD and higher rates of POCI at 3 months compared with the control group
2	Butz et al. [13]	2023	RCT	60 (31 vs. 29)	Paper-and-pencil cognitive training	No cognitive training (standard cardiac rehabilitation)	Approx. 15 days	3 months	SF-36, CFQ	SF-36 mental component summary: η^2^ = 0.102	The cognitive training group demonstrated significantly greater improvements in global HRQoL, overall SF-36 scores, and perceived health change, compared with the control group
3	Butz et al. [14]	2023	RCT	58 (30 vs. 28)	Paper-and-pencil cognitive training	No cognitive training (standard cardiac rehabilitation)	36 min/day, 6 days/week for 3 weeks	12 months	Neuropsychological tests, SF-36, CFQ	POCD incidence: OR = 2.43	Significantly greater improvements in HRQoL were observed in the cognitive training group compared with the control group
4	Butz et al. [15]	2025	RCT	39 (18 vs. 21)	Paper-and-pencil cognitive training	Standard cardiac rehabilitation only	36 min/day, 6 days/week for 3 weeks	3 and 12 months	Neuropsychological tests, SF-36, HADS, MRI for WML/SCI	SF-36 mental component summary: η^2^ = 0.185 (3 months)	The cognitive training group showed greater improvements in cognition and HRQoL at 3 months than the control group, independent of the presence of WML and SCI

Abbreviations: RCT (Randomized Controlled Trial), POCD (Postoperative Cognitive Dysfunction), POCI (Postoperative Cognitive Improvement (POCI), HRQoL (Health-Related Quality of Life), SF-36 (Short Form 36 Health Survey), CFQ (Cognitive Failure Questionnaire), HADS (Hospital Anxiety and Depression Scale), WML (White Matter Lesions), and SCI (Subclinical Cerebral Ischemia). Effect sizes are reported as odds ratios (OR) for dichotomous outcomes and eta-squared (η^2^) for continuous outcomes.

## Data Availability

No new data were generated or analyzed in this study. Therefore, data sharing is not applicable to this article.

## Supplementary Materials

The following supporting information can be downloaded at: https://www.mdpi.com/article/10.3390/jcm15010370/s1. File S1: Search strategy.

## Abbreviations

The following abbreviations are used in this manuscript:

RCT	Randomized Controlled Trial
POCD	Postoperative Cognitive Dysfunction
POCI	Postoperative Cognitive Improvement
HRQoL	Health-Related Quality of Life
SF-36	Short Form 36 Health Survey
CFQ	Cognitive Failures Questionnaire
WML	White Matter Lesions
SCI	Subclinical Cerebral Ischemia
AVR	Aortic Valve Replacement
TAVR	Transcatheter Aortic Valve Replacement
MVR	Mitral Valve Replacement
PRISMA	Preferred Reporting Items for Systematic Reviews and Meta-Analyses

## References

[1] Cicekcioglu F., Ozen A., Tuluce H., Tutun U., Parlar A.I., Kervan U., Karakas S., Katircioglu S.F. (2008). Neurocognitive functions after beating heart mitral valve replacement without cross-clamping the aorta. J. Card. Surg..

[2] Greaves D., Psaltis P.J., Ross T.J., Davis D., Smith A.E., Boord M.S., Keage H.A. (2019). Cognitive outcomes following coronary artery bypass grafting: A systematic review and meta-analysis of 91,829 patients. Int. J. Cardiol..

[3] Fakin R., Zimpfer D., Sodeck G.H., Rajek A., Mora B., Dumfarth J., Grimm M., Czerny M. (2012). Influence of temperature management on neurocognitive function in biological aortic valve replacement. A prospective randomized trial. J. Cardiovasc. Surg..

[4] Oldham M.A., Vachon J., Yuh D., Lee H.B. (2018). Cognitive outcomes after heart valve surgery: A systematic review and meta-analysis. J. Am. Geriatr. Soc..

[5] Patel N., Minhas J.S., Chung E.M. (2015). Risk factors associated with cognitive decline after cardiac surgery: A systematic review. Cardiovasc. Psychiatry Neurol..

[6] van Harten A.E., Scheeren T.W., Absalom A.R. (2012). A review of postoperative cognitive dysfunction and neuroinflammation associated with cardiac surgery and anaesthesia. Anaesthesia.

[7] Martin M., Clare L., Altgassen A.M., Cameron M.H., Zehnder F. (2011). Cognition-based interventions for healthy older people and people with mild cognitive impairment. Cochrane Database Syst. Rev..

[8] Kelly M.E., Loughrey D., Lawlor B.A., Robertson I.H., Walsh C., Brennan S. (2014). The impact of cognitive training and mental stimulation on cognitive and everyday functioning of healthy older adults: A systematic review and meta-analysis. Ageing Res. Rev..

[9] Zhang R., Zhu C.M., Chen S.M., Tian F.M., Huang P.M., Chen Y.M. (2024). Effects of cognitive training on cognitive function in patients after cardiac surgery: A systematic review and meta-analysis of randomized controlled trials. Medicine.

[10] Zhao L., Guo Y., Zhou X., Mao W., Li L. (2023). Preoperative cognitive training improves postoperative cognitive function: A meta-analysis and systematic review of randomized controlled trials. Front. Neurol..

[11] Moher D., Liberati A., Tetzlaff J., Altman D.G., The PRISMA Group (2009). Preferred reporting items for systematic reviews and meta-analyses: The PRISMA statement. J. Clin. Epidemiol..

[12] Butz M., Gerriets T., Sammer G., El-Shazly J., Tschernatsch M., Huttner H.B., Braun T., Boening A., Mengden T., Choi Y.-H. (2022). Effects of postoperative cognitive training on neurocognitive decline after heart surgery: A randomized clinical trial. Eur. J. Cardiothorac. Surg..

[13] Butz M., Gerriets T., Sammer G., El-Shazly J., Tschernatsch M., Schramm P., Doeppner T.R., Braun T., Boening A., Mengden T. (2023). The impact of postoperative cognitive training on health-related quality of life and cognitive failures in daily living after heart valve surgery: A randomized clinical trial. Brain Behav..

[14] Butz M., Gerriets T., Sammer G., El-Shazly J., Tschernatsch M., Braun T., Meyer R., Schramm P., Doeppner T.R., Böning A. (2023). Twelve-month follow-up effects of cognitive training after heart valve surgery on cognitive functions and health-related quality of life: A randomised clinical trial. Open Heart.

[15] Butz M., Gerriets T., Sammer G., El-Shazly J., Braun T., Sünner L., Meyer R., Tschernatsch M., Schramm P., Gerner S.T. (2025). The impact of white matter lesions and subclinical cerebral ischemia on postoperative cognitive training outcomes after heart valve surgery: A randomized clinical trial. J. Neurol. Sci..

[16] Kadoi Y., Kawauchi C., Ide M., Kuroda M., Takahashi K., Saito S., Fujita N., Mizutani A. (2011). Preoperative depression is a risk factor for postoperative short-term and long-term cognitive dysfunction in patients with diabetes mellitus. J. Anesth..

[17] Bekker A., Lee C., de Santi S., Pirraglia E., Zaslavsky A., Farber S., Haile M., de Leon M.J. (2010). Does mild cognitive impairment increase the risk of developing postoperative cognitive dysfunction?. Am. J. Surg..

[18] Berger M., Terrando N., Smith S.K., Browndyke J.N., Newman M.F., Mathew J.P. (2018). Neurocognitive function after cardiac surgery: From phenotypes to mechanisms. Anesthesiology.

[19] Ranucci L., Brischigiaro L., Mazzotta V., Anguissola M., Menicanti L., Bedogni F., Ranucci M. (2024). Neurocognitive function in procedures correcting severe aortic valve stenosis: Patterns and determinants. Front. Cardiovasc. Med..

[20] Mozolic J.L., Hayasaka S., Laurienti P.J. (2010). A cognitive training intervention increases resting cerebral blood flow in healthy older adults. Front. Hum. Neurosci..

[21] Hooks B.M., Chen C. (2020). Circuitry underlying experience-dependent plasticity in the mouse visual system. Neuron.

[22] Appelbaum L.G., Shenasa M.A., Stolz L., Daskalakis Z. (2023). Synaptic plasticity and mental health: Methods, challenges and opportunities. Neuropsychopharmacology.

[23] Zotey V., Andhale A., Shegekar T., Juganavar A. (2023). Adaptive neuroplasticity in brain injury recovery: Strategies and insights. Cureus.

[24] Chatterjee D., Hegde S., Thaut M. (2021). Neural plasticity: The substratum of music-based interventions in neurorehabilitation. NeuroRehabilitation.

[25] Cutuli D., Landolfo E., Petrosini L., Gelfo F. (2022). Environmental enrichment effects on the brain-derived neurotrophic factor expression in healthy condition, Alzheimer’s disease, and other neurodegenerative disorders. J. Alzheimers Dis..

[26] Park D.C., Bischof G.N. (2013). The aging mind: Neuroplasticity in response to cognitive training. Dialog. Clin. Neurosci..

[27] Jiang Y., Xie Y., Fang P., Shang Z., Chen L., Zhou J., Yang C., Zhu W., Hao X., Ding J. (2024). Cognitive training for reduction of delirium in patients undergoing cardiac surgery: A randomized clinical trial. JAMA Netw. Open.

[28] Kainz E., Juilfs N., Harler U., Kahl U., Mewes C., Zöllner C., Fischer M. (2023). The impact of cognitive reserve on delayed neurocognitive recovery after major non-cardiac surgery: An exploratory substudy. Front. Aging Neurosci..

