# Supplementary material for: The Effect of Cognitive Training After Heart Valve Surgery: A Systematic Review
Source: J Clin Med. 2026 Jan 4;15(1):370. doi: 10.3390/jcm15010370 (PMC12786547; doi:10.3390/jcm15010370)
Supplement: Supplementary file 1 [file jcm-15-00370-s001.zip › jcm-4063202-supplementary.pdf]

Search Strategy

This systematic review employed a structured search across four electronic databases (PubMed, Embase, the Cochrane Library, and Scopus) from database inception to June 19, 2025. Searches were restricted to English-language human studies. A combination of database-specific subject headings and free-text keywords was used to maximize the capture of relevant records. Two reviewers independently screened the articles, and a third reviewer resolved discrepancies.

1. Identification Stage (Database Search Overview)

A total of 1506 records were identified through database searching:

- PubMed (n = 369)
- Cochrane Library (n = 334)
- Embase (n = 447)
- Scopus (n = 686)

Duplicate records were removed prior to screening. EndNote X9 automatic duplicate detection was followed by manual validation. A total of 330 duplicate items were removed. No records were excluded by automation tools or for other reasons at this stage, resulting in 1506 records entering the screening phase.

2. Screening Stage (Title and Abstract Review)

A total of 1506 records underwent title and abstract screening. Of these, 1476 records were excluded for not meeting the inclusion criteria. Thirty (30) full-text articles were sought for retrieval, and all were successfully retrieved (n = 30).

3. Eligibility Stage (Full-text Assessment)

Among the 30 full-text articles assessed for eligibility, 26 were excluded for the

following reasons:

- Not focused on patients after heart valve surgery (n = 16);
- Not related to cognitive training (n = 9);
- Review article (n = 1).

Following application of the eligibility criteria, four (4) studies were deemed suitable for inclusion in the final synthesis. All four were randomized controlled trials.

4. Included Studies (Final Selection)

Final included studies (n = 4):

A total of four studies were finally included (n = 4), all of which investigated cognitive training interventions following heart valve surgery.

5. Database-specific Search Syntax (Reproducible)

A comprehensive literature search was conducted across four electronic databases: PubMed, Embase, Cochrane Library and Scopus.

PubMed:

The search strategy used was as follows:

(("valve surgery"[All Fields] OR "heart surgery"[All Fields]) AND ("cognitive"[All Fields] OR "cognition"[All Fields])) AND (fft[Filter])

Embase:

The Embase search included both exploded subject headings and free-text terms:

#1: ('valve surgery' OR 'heart surgery'/exp OR 'heart surgery') AND ('cognitive' OR 'cognition'/exp OR 'cognition') AND ('valve surgery':ti,ab,kw OR 'heart surgery':ti,ab,kw) AND ('cognitive':ti,ab,kw OR 'cognition':ti,ab,kw) AND [english]/lim AND [humans]/lim

Cochrane Library:

The search was conducted using the following terms in All Text:

Trials matching ("valve surgery" OR "heart surgery") AND ("cognitive" OR "cognition") in All Text - (Word variations have been searched)

Scopus:

The search was conducted using the TITLE-ABS-KEYfield, which includes the Title, Abstract, and Keywords.

TITLE-ABS-KEY (('valve AND surgery' OR 'heart AND surgery') AND ('cognitive' OR 'cognition')) AND (LIMIT-TO (LANGUAGE , "English")) AND (LIMIT-TO (EXACTKEYWORD , "Human") OR LIMIT-TO (EXACTKEYWORD , "Humans"))

PRISMA Checklist

Section and Topic	Item #	Checklist item	Location where item is reported
**TITLE**			
Title	1	Identify the report as a systematic review.	Line2
**ABSTRACT**			
Abstract	2	See the PRISMA 2020 for Abstracts checklist.	Line 13-30
**INTRODUCTION**			
Rationale	3	Describe the rationale for the review in the context of existing knowledge.	Line 35-56
Objectives	4	Provide an explicit statement of the objective(s) or question(s) the review addresses.	Line 57-58
**METHODS**			
Eligibility criteria	5	Specify the inclusion and exclusion criteria for the review and how studies were grouped for the syntheses.	Line 78-88
Information sources	6	Specify all databases, registers, websites, organisations, reference lists and other sources searched or consulted to identify studies. Specify the date when each source was last searched or consulted.	Line 63-68
Search strategy	7	Present the full search strategies for all databases, registers and websites, including any filters and limits used.	Line 63-68

Section and Topic	Item #	Checklist item	Location where item is reported
Selection process	8	Specify the methods used to decide whether a study met the inclusion criteria of the review, including how many reviewers screened each record and each report retrieved, whether they worked independently, and if applicable, details of automation tools used in the process.	Line 78-88
Data collection process	9	Specify the methods used to collect data from reports, including how many reviewers collected data from each report, whether they worked independently, any processes for obtaining or confirming data from study investigators, and if applicable, details of automation tools used in the process.	Line 91-96
Data items	10a	List and define all outcomes for which data were sought. Specify whether all results that were compatible with each outcome domain in each study were sought (e.g. for all measures, time points, analyses), and if not, the methods used to decide which results to collect.	Line 91-96
	10b	List and define all other variables for which data were sought (e.g. participant and intervention characteristics, funding sources). Describe any assumptions made about any missing or unclear information.	Line 91-96
Study risk of bias assessment	11	Specify the methods used to assess risk of bias in the included studies, including details of the tool(s) used, how many reviewers assessed each study and whether they worked independently, and if applicable, details of automation tools used in the process.	Line 98-104
Effect measures	12	Specify for each outcome the effect measure(s) (e.g. risk ratio, mean difference) used in the synthesis or presentation of results.	Line 98-104
Synthesis methods	13a	Describe the processes used to decide which studies were eligible for each synthesis (e.g. tabulating the study intervention characteristics and comparing against the planned groups for each synthesis (i	N/A

Section and Topic	Item #	Checklist item	Location where item is reported
		tem #5)).	
	13b	Describe any methods required to prepare the data for presentation or synthesis, such as handling of missing summary statistics, or data conversions.	N/A
	13c	Describe any methods used to tabulate or visually display results of individual studies and syntheses.	N/A
	13d	Describe any methods used to synthesize results and provide a rationale for the choice(s). If meta-analysis was performed, describe the model(s), method(s) to identify the presence and extent of statistical heterogeneity, and software package(s) used.	N/A
	13e	Describe any methods used to explore possible causes of heterogeneity among study results (e.g. subgroup analysis, meta-regression).	N/A
	13f	Describe any sensitivity analyses conducted to assess robustness of the synthesized results.	N/A
Reporting bias assessment	14	Describe any methods used to assess risk of bias due to missing results in a synthesis (arising from reporting biases).	Line 98-104
Certainty assessment	15	Describe any methods used to assess certainty (or confidence) in the body of evidence for an outcome.	Line 98-104
**RESULTS**			
Study selection	16a	Describe the results of the search and selection process, from the number of records identified in the search to the number of studies included in the review, ideally using a flow diagram.	Line 107-113

Section and Topic	Item #	Checklist item	Location where item is reported
	16b	Cite studies that might appear to meet the inclusion criteria, but which were excluded, and explain why they were excluded.	Line 107-113
Study characteristics	17	Cite each included study and present its characteristics.	Line 107-127
Risk of bias in studies	18	Present assessments of risk of bias for each included study.	Line 116-127
Results of individual studies	19	For all outcomes, present, for each study: (a) summary statistics for each group (where appropriate) and (b) an effect estimate and its precision (e.g. confidence/credible interval), ideally using structured tables or plots.	Line 130-196
Results of syntheses	20a	For each synthesis, briefly summarise the characteristics and risk of bias among contributing studies.	Line 130-196
	20b	Present results of all statistical syntheses conducted. If meta-analysis was done, present for each the summary estimate and its precision (e.g. confidence/credible interval) and measures of statistical heterogeneity. If comparing groups, describe the direction of the effect.	Line 130-196
	20c	Present results of all investigations of possible causes of heterogeneity among study results.	Line 130-196
	20d	Present results of all sensitivity analyses conducted to assess the robustness of the synthesized results.	Line 130-196
Reporting bi	21	Present assessments of risk of bias due to missing results (arising from reporting biases) for each sy	Line 116

Section and Topic	Ite m #	Checklist item	Location where item is reported
ases		nthesis assessed.	-127
Certainty of evidence	22	Present assessments of certainty (or confidence) in the body of evidence for each outcome assessed.	Line 116 -127
**DISCUSSION**			
Discussion	23a	Provide a general interpretation of the results in the context of other evidence.	Line 202 -260
	2 3 b	Discuss any limitations of the evidence included in the review.	Line 202 -260
	23c	Discuss any limitations of the review processes used.	Line 202 -260
	2 3 d	Discuss implications of the results for practice, policy, and future research.	Line 202 -260
**OTHER INFORMATION**			
Registration and protocol	24a	Provide registration information for the review, including register name and registration number, or state that the review was not registered.	Line 72-76
	2 4 b	Indicate where the review protocol can be accessed, or state that a protocol was not prepared.	Line 72-76
	24c	Describe and explain any amendments to information provided at registration or in the protocol.	Line 72-

Section and Topic	Item #	Checklist item	Location where item is reported
			76
Support	25	Describe sources of financial or non-financial support for the review, and the role of the funders or sponsors in the review.	Line 270
Competing interests	26	Declare any competing interests of review authors.	Line 276
Availability of data, code and other materials	27	Report which of the following are publicly available and where they can be found: template data collection forms; data extracted from included studies; data used for all analyses; analytic code; any other materials used in the review.	Line 273, 274
